# Catching the Reversible Formation and Reactivity of Surface Defective Sites in Metal–Organic Frameworks: An Operando Ambient Pressure-NEXAFS Investigation

**DOI:** 10.1021/acs.jpclett.1c02585

**Published:** 2021-09-16

**Authors:** Luca Braglia, Francesco Tavani, Silvia Mauri, Raju Edla, Damjan Krizmancic, Alessandro Tofoni, Valentina Colombo, Paola D’Angelo, Piero Torelli

**Affiliations:** † CNR-Istituto Officina dei Materiali, TASC, 34149 Trieste, Italy; ‡ Dipartimento di Chimica, Università di Roma “La Sapienza”, Piazzale Aldo Moro 5, 00185 Rome, Italy; § Institute for Photon Science and Synchrotron Radiation, Karlsruhe Institute of Technology, D-76344, Eggenstein-Leopoldshafen, Germany; ∥ Dipartimento di Chimica, Università degli Studi di Milano, Via Golgi 19, 20133 Milan, Italy

## Abstract

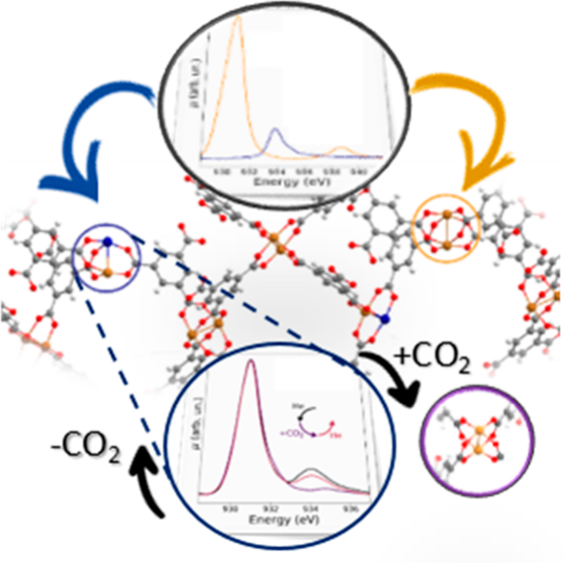

In this work, we apply for the first time ambient pressure operando soft X-ray absorption spectroscopy (XAS) to investigate the location, structural properties, and reactivity of the defective sites present in the prototypical metal–organic framework HKUST-1. We obtained direct evidence that Cu^+^ defective sites form upon temperature treatment of the powdered form of HKUST-1 at 160 °C and that they are largely distributed on the material surface. Further, a thorough structural characterization of the Cu^+^/Cu^2+^ dimeric complexes arising from the temperature-induced dehydration/decarboxylation of the pristine Cu^2+^/Cu^2+^ paddlewheel units is reported. In addition to characterizing the surface defects, we demonstrate that CO_2_ may be reversibly adsorbed and desorbed from the surface defective Cu^+^/Cu^2+^ sites. These findings show that ambient pressure soft-XAS, combined with state-of-the-art theoretical calculations, allowed us to shed light on the mechanism involving the decarboxylation of the paddlewheel units on the surface to yield Cu^+^/Cu^2+^ complexes and their reversible restoration upon exposure to gaseous CO_2._

Metal–organic frameworks (MOFs) are emerging nanoporous materials obtained from the binding of polydentate organic molecules (the linkers) to metal ions or clusters (the nodes) generating three-dimensional structures featuring pores with nanosized apertures and ultrahigh internal surface areas (up to 10 000 m^2^ g^–1^).^1^ Tailor-made MOFs for specific applications, such as highly selective adsorbents for target molecules, can be obtained by engineering the coordination of the linkers to the metal nodes.^2^ The combination of compositional modularity, synthetic ease, and multifunctional properties have led to the introduction of MOFs into a wide range of application fields, such as gas sorption (separation and storage),^3−8^ catalysis,^9,10,3^ photocatalysis,^11^ sensing,^12^ heat transformation,^13^ and drug delivery.^14,15^ Within these applications, MOFs have arisen as promising materials for the selective and reversible capture of CO_2_,^16−18^ a crucial environmental issue, and the introduction of coordinatively active sites (CUSs) and their postsynthetic functionalization have been found to be very powerful approaches to improve CO_2_ uptake.^19−21^ CUSs are thus homogeneously dispersed within the framework and available as defined, isolated, single active sites for gas adsorption and catalysis, with an unprecedented mimicking of enzyme behavior.^3^ However, it is still unclear whether the MOF catalytic and absorption properties are only related to the CUSs present in the “perfect” crystal structure or are enhanced, or even due to the presence of structural defects within the MOF itself. In this context, an archetypal example, widely studied for both its activity at the Cu-open metal sites and the defect-engineering of its crystal structure, is the well-known HKUST-1 (denoted also as Cu_3_(BTC)_2_, BTC = benzene-1,3,5-tricarboxilate, Figure S1).^22^

However, despite the long history of research on the causes and nature of the mixed valence defective dimer sites in HKUST-1 (a brief summary is reported in the Supporting Information), full agreement on their formation, structure, and reactivity has not yet been reached.

While hard X-ray absorption spectroscopy (XAS) at the Cu K-edge has been widely employed to characterize the local structure and reactivity of the HKUST-1 copper sites,^23−26^ the application of soft-XAS at the Cu L_2,3_-edges has been severely limited by the need for high vacuum conditions and tailored experimental set-ups. In a very recent development, specific cells have been designed that allow soft-XAS experiments to be carried out at atmospheric pressure under operando conditions (AP-NEXAFS) (technique details are in the Supporting Information). In this case, soft-XAS is operated in the total electron yield (TEY) detection mode, which renders the technique surface sensitive, owing to the low electron escape depth which limits the thickness of the probed sample to a few atomic layers below the surface. The newly developed AP-NEXAFS technique is a powerful method to unveil the nature of CUSs in MOFs during adsorption experiments or even catalytic reactions, since its surface sensitivity (<10 nm) is a crucial feature to thoroughly characterize the defective sites that occur on the surface of the investigated material.

Figure 1a shows the comparison between the Cu L_3_-edge XAS spectrum of the pristine HKUST-1 collected in He flux (50 mL/min, 1 bar) at RT and that of the same sample exposed to a He flux at 160 °C for 10 min. The spectrum collected at RT shows an intense asymmetric peak at 930.7 eV (peak A in Figure 1a) with a broad shoulder at 931.9 eV (peak B, Figure 1a) that disappears after the thermal treatment. Moreover, the temperature increase leads to the appearance of a new feature at 934.1 eV (peak C, Figure 1a) that is located at the same energy position as the white line of the Cu L_3_-edge spectrum of the Cu_2_O reference sample (Figure 1b). The disappearance of peak B in the spectrum collected at 160 °C might be due to the change in coordination of the Cu^2+^ sites upon temperature treatment, while the appearance of feature C is consistent with the formation of Cu^+^ species upon reduction of the Cu^2+^ surface ions. Note that the main transition of the Cu_2_O spectrum has an asymmetric shape with a pronounced tail toward higher energy, while peak C in the spectrum of the thermally treated HKUST-1 is more symmetric. Formally, the Cu^+^ ion has a d^10^ electronic configuration, and consequently the 2p → 3d transition resulting in peak C should not be observed since all the d states are occupied. However, the geometry of the Cu^+^ sites can give rise to a partial 3d character in the empty density of states as in the known case of the linear Cu_2_O oxide.^27,28^ Moreover, both spectra collected at RT and at 160 °C show an additional broad peak at about 938.4 eV (peaks D, Figure 1a), which is known to be related to the 2p → 4s electronic transition in the Cu^2+^ ions.^27^

**Figure 1:**
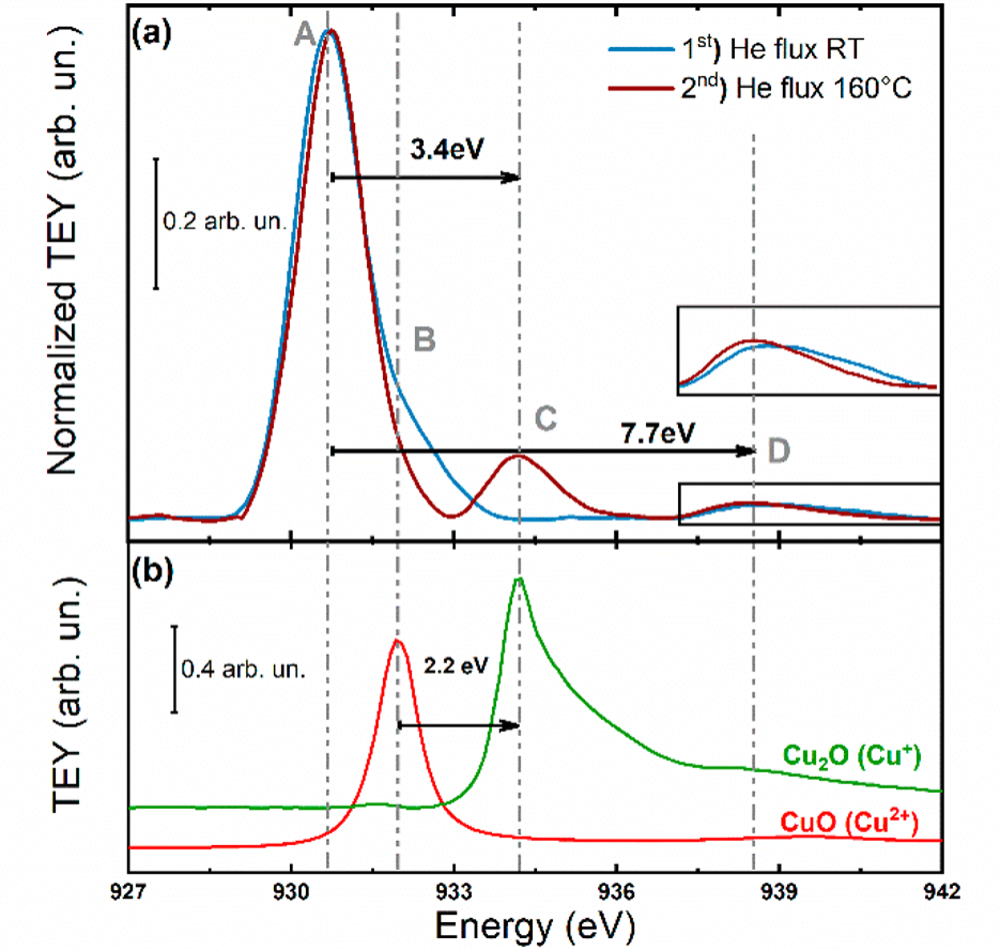
(a) Cu L_3_-edge AP-NEXAFS spectra of HKUST-1 collected in 1 bar flux of He at RT (blue line) and in He at 160 °C (brown line). Experimental peak maxima are indicated by dashed lines with a references letter A,B,C and D. (b) Cu L_3_-edge AP-NEXAFS spectra of Cu_2_O (green line) and CuO (red line). The spectra in (b) are vertically shifted.

Previous investigations on HKUST-1 have disclosed that temperature annealing leads to the dehydration of the Cu^2+^/Cu^2+^ paddlewheel units present in the pristine MOF and to the formation of Cu^+^ species. The presence of partially reduced Cu^+^/Cu^2+^ dimeric sites has been observed by CO-probed FTIR and XPS,^29−33^ and a broad discussion has been established in the literature on the origin of the Cu^+^ species in HKUST-1. In particular, two different hypotheses have been made: the former suggests that the Cu^+^ ions originate from amorphous Cu_2_O impurities that are formed upon heating at high temperature (e.g., 350 °C),^29^ and the latter suggests Cu^+^/Cu^2+^ dimeric sites originate from the Cu^2+^ ions in the MOF framework either by reduction of defective clusters or by reduction of Cu^2+^ cations in perfectly coordinated paddlewheels.^32,33^ Consequently, in our experimental conditions, it appears reasonable to hypothesize that the temperature treatment provokes both the dehydration of the pristine Cu^2+^/Cu^2+^ paddlewheel units and the formation of defective Cu^+^/Cu^2+^ sites on the surface of HKUST-1. Note that the soft-XAS spectra collected in the TEY detection mode probe only the first few atomic layers from the surface, and consequently the effects we are reporting are mainly confined on the surface of our material. The Cu^+^ surface defective sites observed in our AP-NEXAFS spectrum are unlikely due to Cu_2_O impurities since we carried out a mild annealing treatment at 160 °C. In order to support this view, we also measured the Cu L_2,3_-edge spectrum of CuO in a He flux increasing the temperature up to 210 °C. In this case, no Cu^+^ ions were formed on the surface as evidenced by the absence of peak C in the red spectrum of Figure S2, while only exposure of CuO to a flux of CO gas, acting as a reducing agent, led to the reduction of Cu^2+^ to Cu^+^ (see spectra in brown in Figures 1b and S2). Moreover, the PXRD pattern confirms the absence of both CuO and Cu_2_O impurities in the HKUST-1 at RT (Figure S3). In order to investigate the structure of the defective sites formed upon heating and to provide a conclusive characterization of all of the features present in the HKUST-1 NEXAFS spectra, we performed a theoretical analysis using the FDMNES code.^34^ In the first step, the Cu L_2,3_-edge spectra of the CuO and Cu_2_O reference samples were calculated in order to benchmark the theoretical framework (see Table S1), and the results are shown in Figure S4. The theoretical spectra are in good agreement with the experimental data shown in Figure 1b, and both the asymmetric shape of the main absorption edge of the Cu_2_O spectrum and the more symmetric shape of the white line of the CuO experimental data are properly reproduced by the theoretical calculations (see Figures 1b and S4).

In the second step of our analysis, the Cu L_2,3_-edge spectrum of the pristine MOF at RT was calculated starting from the crystallographic structure of HKUST-1.^35^ In this structure, the Cu^2+^ ions are coordinated by five oxygen atoms in a square pyramidal configuration at a Cu–O distance of 1.852 Å with the apical oxygen atom belonging to a water molecule placed at 2.207 Å from the Cu^2+^ ion. The full list of structural parameters used in the theoretical calculations are listed in Table S2. The comparison between the theoretical and experimental Cu L_3_-edge spectra of the as-synthesized hydrated MOF sample is reported in Figure S5 along with the associated dimeric cluster. The experimental and theoretical curves are in very good agreement as far as the energy position and the relative intensity of peaks A and D are concerned, and also peak B, which is mainly associated with the water molecule coordinated in the axial position, is nicely reproduced by the theoretical calculations. In order to uncover the local structural properties of the Cu^2+^ and Cu^+^ species present in the thermally treated HKUST-1 sample, we carried out a thorough analysis of the NEXAFS data. First, the relative abundance between the Cu^+^ sites formed upon thermal induced defect formation and the square planar (SP)-coordinated Cu^2+^ sites was estimated by means of a Voigt function fitting as the ratio of the areas of peaks C and A, as shown in Figure S6. Note that these areas need to be normalized by the cross sections of Cu^+^ and Cu^2+^, and to this aim we have followed the same procedure as described in Fracchia et al.^36^ This analysis led us to estimate the surface concentration of the Cu^+^ sites to be 22.7% and, accordingly, that of the Cu^+^/Cu^2+^ dimers to be approximately 45.4% on the surface (<10 nm) of our sample.

Next, theoretical Cu L_3_-edge spectra were calculated for two distinct models: a Cu^2+^/Cu^2+^ dimer where both metal cations are SP-coordinated, and a Cu^+^/Cu^2+^ dimer arising from the hypothesized oxidative decarboxylation of the former complex where both metal centers are coordinated by three oxygen atoms (Figure 2a). The theoretical Cu L_3_-edge spectra belonging to the Cu^2+^ and Cu^+^ ions present in the Cu^2+^/Cu^2+^ and Cu^+^/Cu^2+^ clusters and weighted by their estimated surface relative abundance are shown in Figure 2b, together with the associated molecular structures. The shape of the theoretical spectrum assigned to the Cu^2+^ cation is fairly symmetric, while that of the Cu^+^ species is skewed toward higher energies similar to the shape of peak C and to the experimental and theoretical Cu L_3_-edge spectra of Cu_2_O. Starting from this result, a theoretical NEXAFS curve has been derived by adding the spectra assigned to the Cu^2+^ cation in the Cu^2+^/Cu^2+^ dimer together with those of the Cu^+^ and Cu^2+^ cations in the Cu^+^/Cu^2+^ complex.

**Figure 2:**
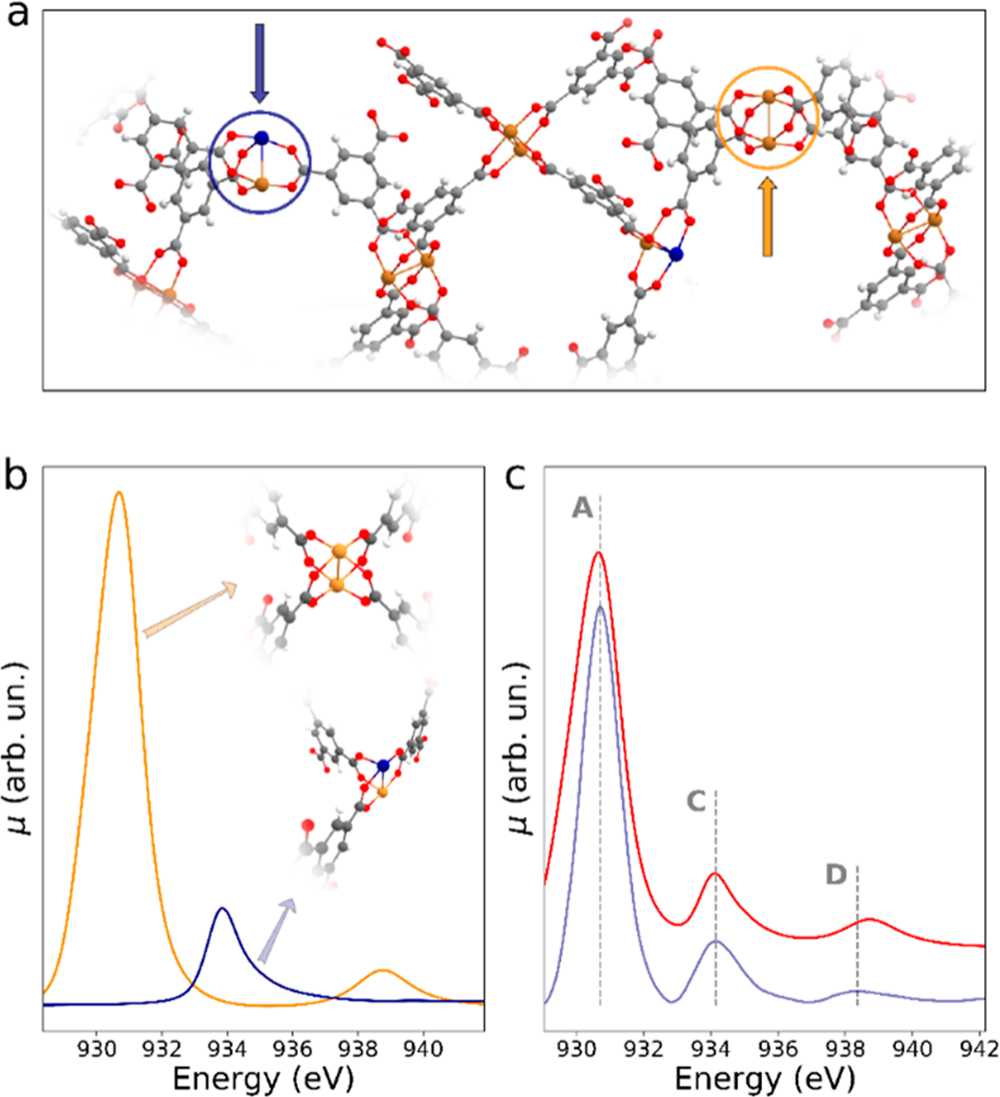
(a) Depiction of the HKUST-1 surface copper sites formed upon treatment of the MOF powder at 160 °C in the He flux. The resulting Cu^2+^/Cu^2+^ and Cu^+^/Cu^2+^ dimeric sites are evidenced by yellow and blue arrows, respectively. (b) Theoretical Cu L_3_-edge spectra simulated for the Cu^2+^ (yellow) and Cu^+^ (blue) cations present in the Cu^2+^/Cu^2+^ and Cu^+^/Cu^2+^ dimers, respectively, and weighted by the estimated relative surface abundance. (c) Comparison between the experimental Cu L_3_-edge spectrum of HKUST-1 collected at 160 °C in He flux (red) and the theoretical spectrum resulting from the weighted sum of the spectra belonging to the Cu^2+^ and Cu^+^ surface species (light purple). Constant energy cuts (dotted grey lines) are drawn in proximity of the experimental maxima of peaks A, C and D.

During this procedure, the spectra were weighted by the previously determined relative surface abundance. The resulting total theoretical spectrum (light purple) is compared to the experimental spectrum (red) of the HKUST-1 collected at 160 °C in Figure 2c. The agreement between the two spectra is very good, and the energy positions and relative intensities of peaks A, C, and D are all correctly reproduced, proving the reliability of the analysis.

Overall, these findings confirm that upon a mild annealing at 160 °C in the He flux the Cu^2+^ sites present in the pristine HKUST-1 are dehydrated with the formation of SP-coordinated clusters, and some of the paddlewheels undergo decarboxylation with the production of partially reduced Cu^+^/Cu^2+^ dimers. The defective sites have been found to be located mostly on the surface as the percentage of reduced copper found in the present study is quite high (22.7%), while in the case of the powder MOF, bulk sensitive techniques have estimated the Cu^+^ species formed upon temperature treatment to be about 3% of the total Cu in the system.^30^

After elucidating the nature of the regular and defective copper sites in the HKUST-1, we investigated the reactivity of the defective sites in the presence of two prototypical gases, namely, CO_2_ and H_2_, that are generally used to promote the oxidation and reduction of metal cations. To this aim, we collected the Cu L_3_-edge spectra of the MOF at 160° fluxing pure He and its mixtures with CO_2_ in a reactor cell containing the material. In Figure 3a, we report the Cu L_3_-edge experimental spectra of HKUST-1 collected at 160 °C in the He flux before, during and after exposure to a 2% flux of CO_2_. One may observe that upon exposure to CO_2_ the intensity of peak C decreases due to the oxidation of Cu^+^ to Cu^2+^ (Figure 1a), while peak B is not restored. This indicates that almost all of the thermally induced Cu^+^/Cu^2+^ defective sites are oxidized by CO_2_, while no water molecules coordinate the Cu^2+^ ions in the apical positions. This further confirms that peak B in the RT spectrum of the HKUST-1 is the fingerprint of the water ligands, and to get a definite proof, the theoretical spectrum calculated for the Cu^2+^ cation in the dehydrated Cu^2+^/Cu^2+^ dimeric complex has been compared to the experimental spectrum obtained after the CO_2_ flux in Figure S8.

**Figure 3:**
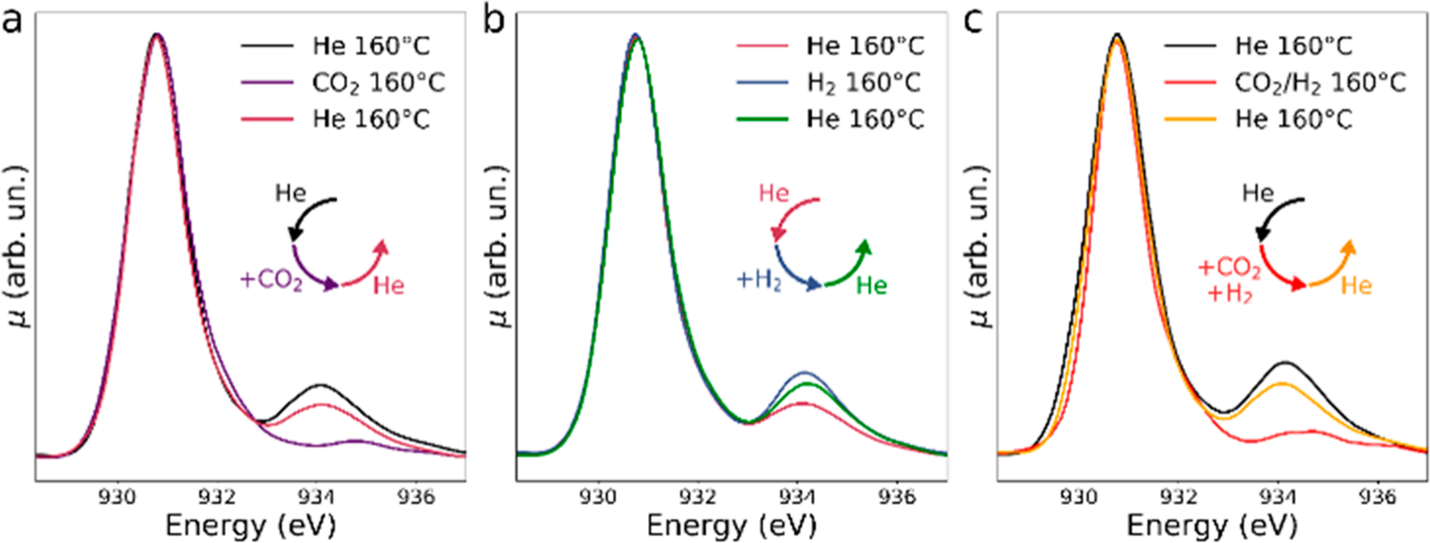
Series of Cu L_3_-edge AP-NEXAFS spectra of HKUST-1: (a) collected at 160 °C in 1 bar of He flux before (black line) and after exposure to CO_2_ (violet line), and in 1 bar of He flux after removal of CO_2_ (dark red line), (b) in 1 bar He flux before (dark red line) and after exposure to H_2_ (blue line), and in 1 bar of He flux after removal of H_2_ (green line), (c) at 160 °C in a 1 bar flux of He before (black line), during (red line) and after exposure to a flux of a gaseous mixture containing CO_2_ (2%) and H_2_ (6%) (orange line). In order to aid the visualization, in each panel the chronological order with which the spectra were measured is portrayed using sets of arrows arranged in circles whose colors match those of the corresponding presented spectra and whose orientations evidence the temporal sequence with which each spectrum was collected.

The agreement between the experimental and theoretical spectra is very good, and the shape and width of peak A are nicely reproduced.

Looking at Figure 3a, one may note that when the CO_2_ flux is interrupted the intensity of peak C is almost completely recovered, showing that the defective Cu^+^/Cu^2+^ sites on the MOF surface are almost completely restored. This finding demonstrates that the oxidation of the Cu^+^ sites in the presence of CO_2_ is a reversible process. Finally, the sample previously exposed to CO_2_ has been fluxed with He containing 6% of H_2_, and the percentage of Cu^+^/Cu^2+^ defective sites further increases, as demonstrated by the intensity of peak C (see Figure 3b).

In order to shed light on the mechanism of the CO_2_ interaction with the MOF, the O K-edge AP-NEXAFS spectra of HKUST-1 at 160 °C before and during the CO_2_ flux were collected (Figure S7). By looking at the O K-edge spectra of the CO and CO_2_ gases reported in Figure S7 for comparison, the decomposition of CO_2_ to CO during the flux on the MOF can be excluded as the main peaks of the π bonds of the CO_2_ gas are clearly present and of the CO gas, that falls at a separate energy, is absent in the O K-edge AP-NEXAFS spectrum of HKUST-1 under CO_2_ flux. All together, these findings suggest that the mechanism of CO_2_ interaction is through a redox-active transition on the metal Cu^+^ defective sites. Figure 4 shows the mechanistic picture we have derived from the temperature-induced surface properties of HKUST-1. In particular, it can be hypothesized that the presence of defective sites in the pristine HKUST-1 enables the partial reduction of the dehydrated Cu^2+^/Cu^2+^ units to Cu^+^/Cu^2+^ dimers upon a temperature treatment at 160 °C and that the Cu^2+^/Cu^2+^ and Cu^+^/Cu^2+^ surface complexes reversibly shuttle between each other in the presence or absence of a CO_2_ external gas flux.

**Figure 4:**
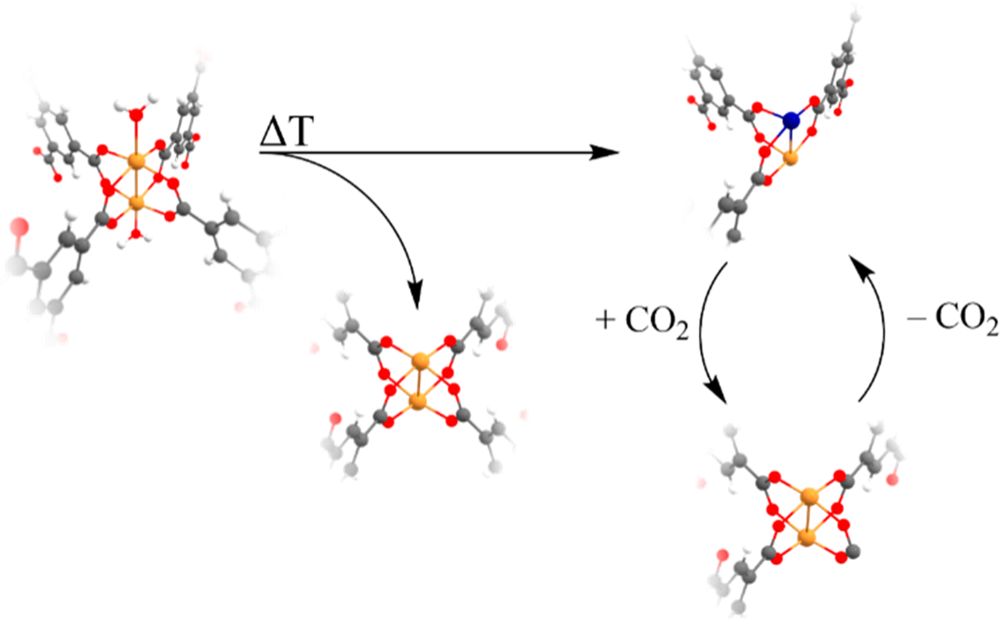
Proposed mechanistic scheme for the investigated surface properties of HKUST-1. The paddlewheel unit of the pristine MOF is converted to the dehydrated Cu^2+^/Cu^2+^ dimer upon temperature treatment at 160 °C, which in turn undergoes a partial decarboxylation to yield a Cu^+^/Cu^2+^ complex. The surface Cu^2+^/Cu^2+^ dimer is reversibly replenished upon exposure of the Cu^+^ sites to CO_2_ in the gas phase.

Finally, in order to investigate the selectivity of the Cu^+^ defective sites in HKUST-1 toward CO_2_ capture, we have monitored the evolution of the Cu L_3_-edge spectrum of the MOF at 160 °C under a flux of He containing a mixture of CO_2_ (2%) and H_2_ (6%). The results are reported in Figure 3c, where it appears that the intensity of peak C is greatly reduced upon flux of gaseous mixture showing that the Cu^+^ sites of the material preferentially interact with CO_2_ undergoing an oxidation process. It is of note that the presence of H_2_ in the flux does not significantly affect such interaction since the Cu L_3_-edge spectra of the sample measured at 160 °C under a He flux containing CO_2_ (2%)/H_2_ (6%) (Figure 3c, red line) and the one containing only CO_2_ (2%) are very similar (Figure 3a, violet line), confirming that the Cu^+^ sites interact easily and preferentially with CO_2_. The CO_2_ capture of the MOF is again proven to be reversible since once the CO_2_ gas flux is interrupted peak C is almost completely recovered (Figure 3c, orange line).

In conclusion, a thorough characterization of the thermally induced properties of the surface Cu sites in the HKUST-1 has been achieved by combining an innovative experimental technique such as AP-NEXAFS with theoretical support. For the first time, the Cu L_3_-edge spectra of the HKUST-1 have been collected at ambient pressure (1 bar) in a temperature range going from RT to 160 °C in different gas environments (He, CO_2_, H_2_, and CO_2_/H_2_). The AP-NEXAFS spectroscopy allowed us to fully unveil the structural properties of the copper sites present in the first layers of HKUST-1, and the unique surface sensitivity of this technique enabled us to prove that defective Cu^+^/Cu^2+^ dimeric sites are largely present on the surface of the investigated material. Within our experimental and theoretical framework, we have clear evidence of the formation of Cu^+^ surface sites upon temperature treatment of the pristine MOF at 160 °C, and we estimate the Cu^+^/Cu^2+^ species to be ca. 45.4% of the total amount of Cu dimers on the surface of the sample. Moreover, we propose that the Cu^+^/Cu^2+^ dimeric units arise from a decarboxylation of dehydrated Cu^2+^/Cu^2+^ paddlewheel units, while the formation of Cu^+^ defective sites is unlikely due to the presence of Cu_2_O impurities in the MOF, as previously suggested,^23^ since the very strong Cu–O bonds contained in the oxide should not be affected by an annealing at 160 °C. Further, we show for the first time that CO_2_ may be fruitfully employed as a probe molecule in the gas phase to study the surface properties of HKUST-1 and reversibly oxidize the temperature-induced Cu^+^ sites. We believe that our results may lead to an increased understanding of the surface properties of HKUST-1 and pave the way for their rational use in processes of interest for catalysis.

## Supporting Information Available

Detailed information about a quick summary in the literature about the defects in HKUST-1, experimental and theoretical soft-XAS, PXRD pattern, defects quantifications (PDF)
