# Supplementary material for: Catching the Reversible Formation and Reactivity of Surface Defective Sites in Metal–Organic Frameworks: An Operando Ambient Pressure-NEXAFS Investigation
Source: J Phys Chem Lett. 2021 Sep 16;12(37):9182–7. doi: 10.1021/acs.jpclett.1c02585 (PMC9282676; doi:10.1021/acs.jpclett.1c02585)
Supplement: Supplementary file 1 — jz1c02585_si_001.pdf [file jz1c02585_si_001.pdf]

Supporting Information of

"Catching the reversible formation and reactivity of surface defective sites in MOFs: an operando Ambient Pressure-NEXAFS investigation"

Luca Braglia[†]*, Francesco Tavani[‡], Silvia Mauri[†], Raju Edla[†,§], Damjan Krizmancic[†], Alessandro Tofoni[‡], Valentina Colombo[‖], Paola D'Angelo[‡], Piero Torelli[†]

AUTHOR ADDRESS

[†] CNR- Istituto Officina dei Materiali, TASC, Trieste, Italy

[‡] Dipartimento di Chimica, Università di Roma "La Sapienza", P.le A. Moro 5, 00185 Roma, Italy

[§] Institute for Photon Science and Synchrotron Radiation, Karlsruhe Institute of Technology, Eggenstein-Leopoldshafen, Germany

[‖]Dipartimento di Chimica, Università degli Studi di Milano, Via Golgi 19, 20133 Milan, Italy

History about Cu^{2+}/Cu^{+} dimers in HKUST-1 systems

In HKUST-1 the inorganic node is based on a paddlewheel unit, which contains Cu^{2+}/Cu^{2+} dimers, resembling the cupric acetate ones. These Cu^{2+} sites are arranged in a square pyramidal configuration where each copper cation is coordinated by four oxygen atoms of the BTC linkers, while a polar solvent molecule (*e.g.* water) may occupy the apical position (Figure S1). CUSs in HKUST-1 can be easily generated by removing the apical water molecules with gentle heating under low pressure, resulting in the chemically activated structure with exposed Cu^{2+} sites available for additional ligands. [1,2,3,4]

Recently, it has been reported that Cu^{+}/Cu^{2+} dimer defects may be created via thermally induced reduction of the Cu^{2+}/Cu^{2+} square planar (SP)-coordinated pairs in HKUST-1 MOF. This process involves a Cu^{2+}-catalyzed oxidative decarboxylation, with the Cu^{2+}/Cu^{2+} units being partially reduced to Cu^{+}/Cu^{2+} dimers, where each metal cation is coordinated by three oxygen atoms.[5,6,7,8]

Further, it has been proposed that upon water vapor exposure the SP Cu^{2+}/Cu^{2+} sites of the Cu-paddle-wheel structure in HKUST-1 may undergo a transformation to Cu^{+}/Cu^{2+} mixed dimers where an hydroxide ion, produced from a heterolitically dissociated water molecule, coordinates one of the Cu^{2+} ions and causes a reduction of the other Cu^{2+} to a Cu^{+} ion. [5,9,34]

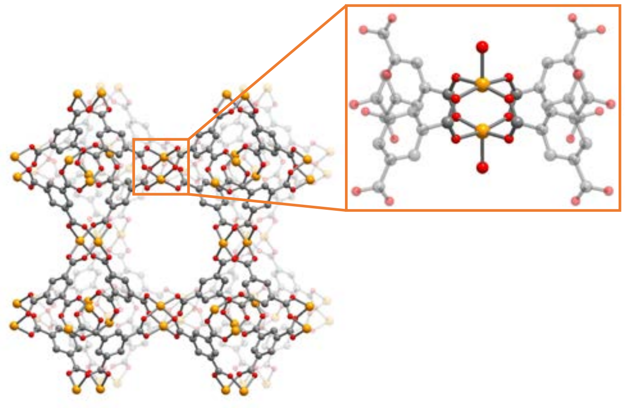

Figure S1. Unit cell of HKUST-1 MOF and zoom on the Cu^{2+}/Cu^{2+} hydrated paddlewheel unit. The Cu^{2+} cations are depicted in orange, while the oxygen and carbon atoms are shown in red and grey respectively.

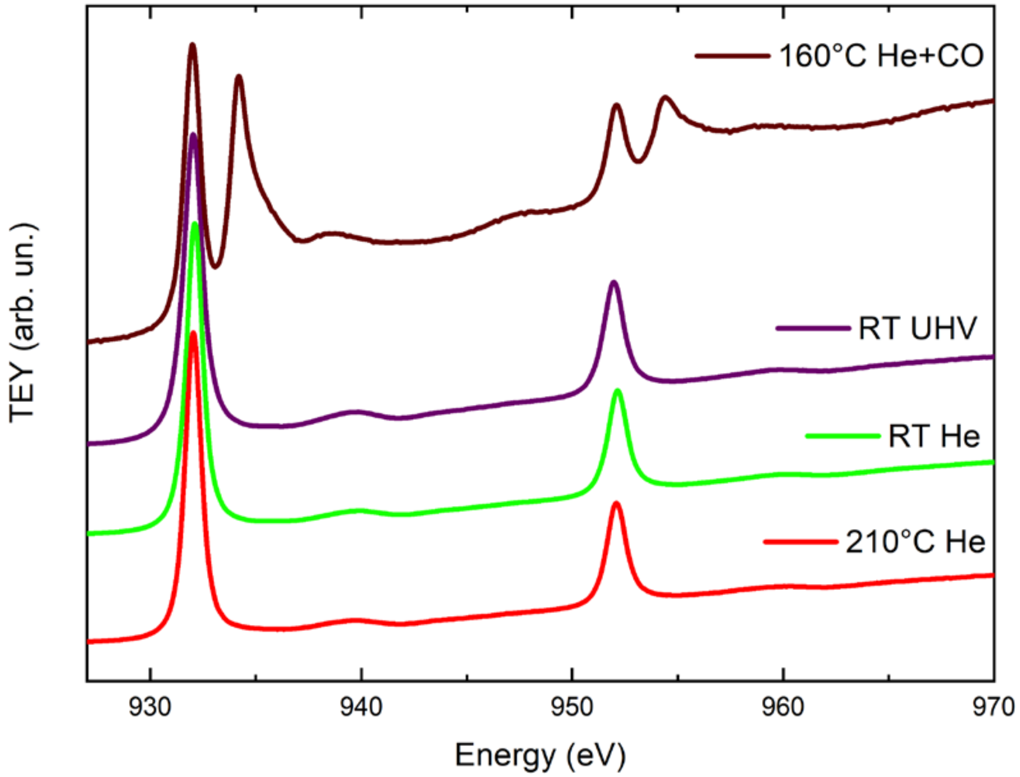

Figure S2. Cu $L_{2,3}$-edge spectra of CuO measured in different conditions: at 160°C in He flux with 2% of CO (brown line), in vacuum at RT (violet line), in He flux at RT (green line), and in He flux at 210°C (red line).

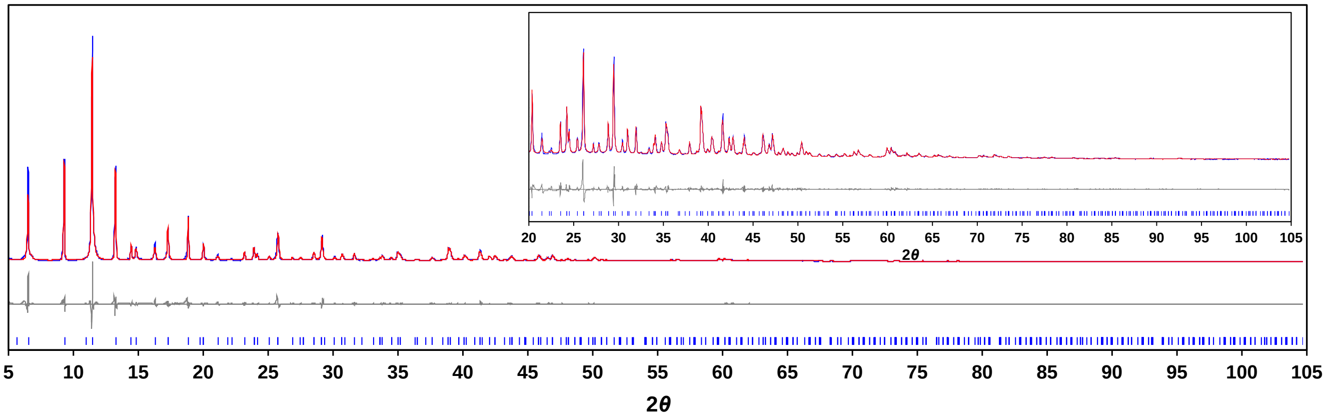

Figure S3. Structureless Le Bail refinement plot for HKUST-1 as such. Blue trace and red trace represent the experimental and calculated diffractograms, respectively. Grey line represents the difference between the experimental and calculated profiles. Blue tick marks are the calculated peak positions. The inset shows an enlargement of the high angle region. R_p and R_{wp}, 0.0864 and 0.1253 respectively, for 5001 data in the 5-105 2θ range.

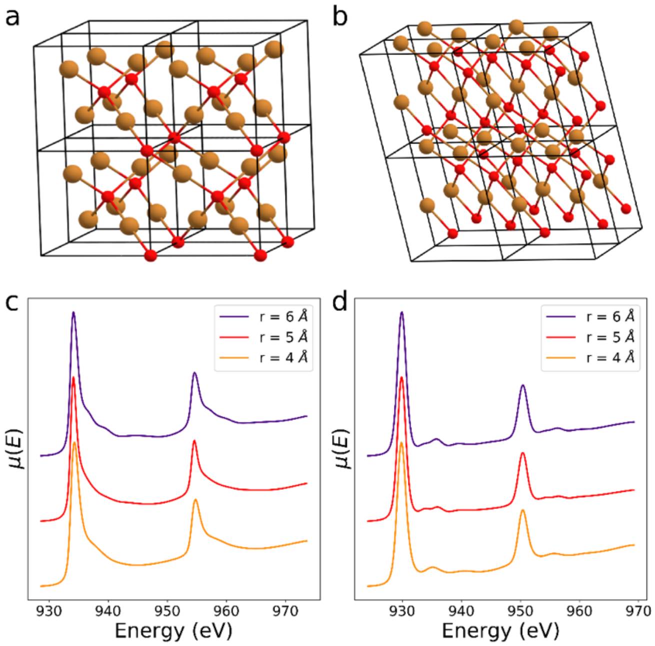

Figure S4. Representative crystallographic structures of Cu_2O and CuO (panels a and b respectively) where the copper cations and oxygen atoms are depicted in orange and red, respectively. Cu $L_{2,3}$-edge theoretical spectra (panels c and d respectively) calculated from the crystal structures shown above by including all of the atoms within cut-off radii of 4, 5 and 6 Å from the Cu photoabsorber.

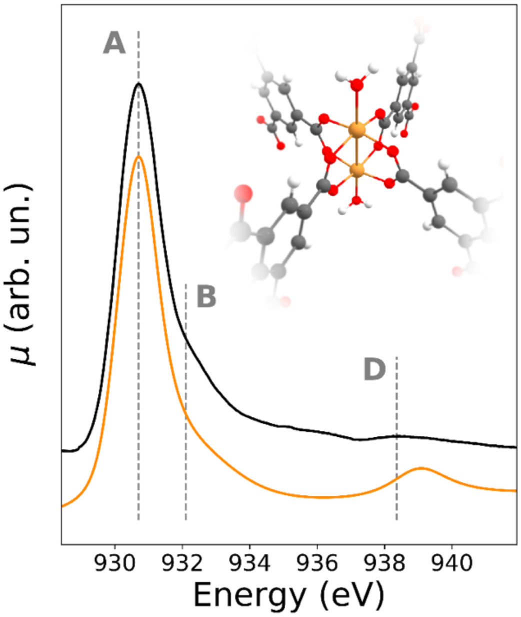

Figure S5. Comparison between the Cu L_3-edge experimental (black) and theoretical (yellow) spectra relative to the pristine HKUST-1 MOF collected at RT. Constant energy cuts (dotted grey lines) are drawn in proximity of the experimental maxima of peaks A, B and D. The associated molecular cluster is also shown, where the Cu^{2+} cation and the oxygen, carbon and hydrogen atoms are depicted in orange, red, black and white, respectively.

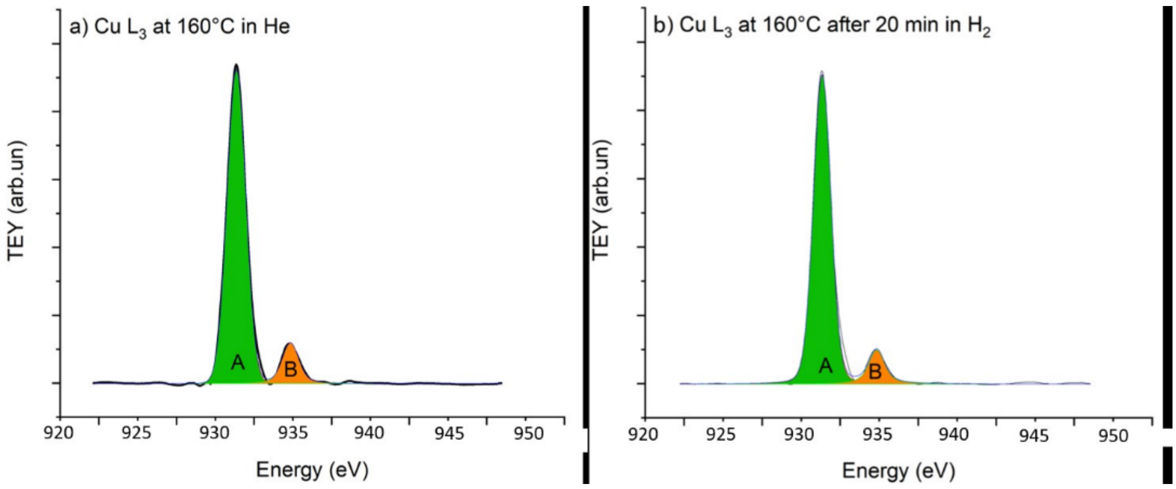

Figure S6. Voigt function fits of the Cu L_3-edge spectrum of the HKUST-1 MOF collected at 160°C in He flux (a) and H_2 flux (b). The area of peak B is 12% (22.7%) and 11% (20.8%) of the total in the two spectra. This ratio provides an estimate of the ratio between the Cu^{+} and Cu^{2+} ions present on the MOF surface, the values in brackets are normalized by the cross sections of Cu^{+} and Cu^{2+}.

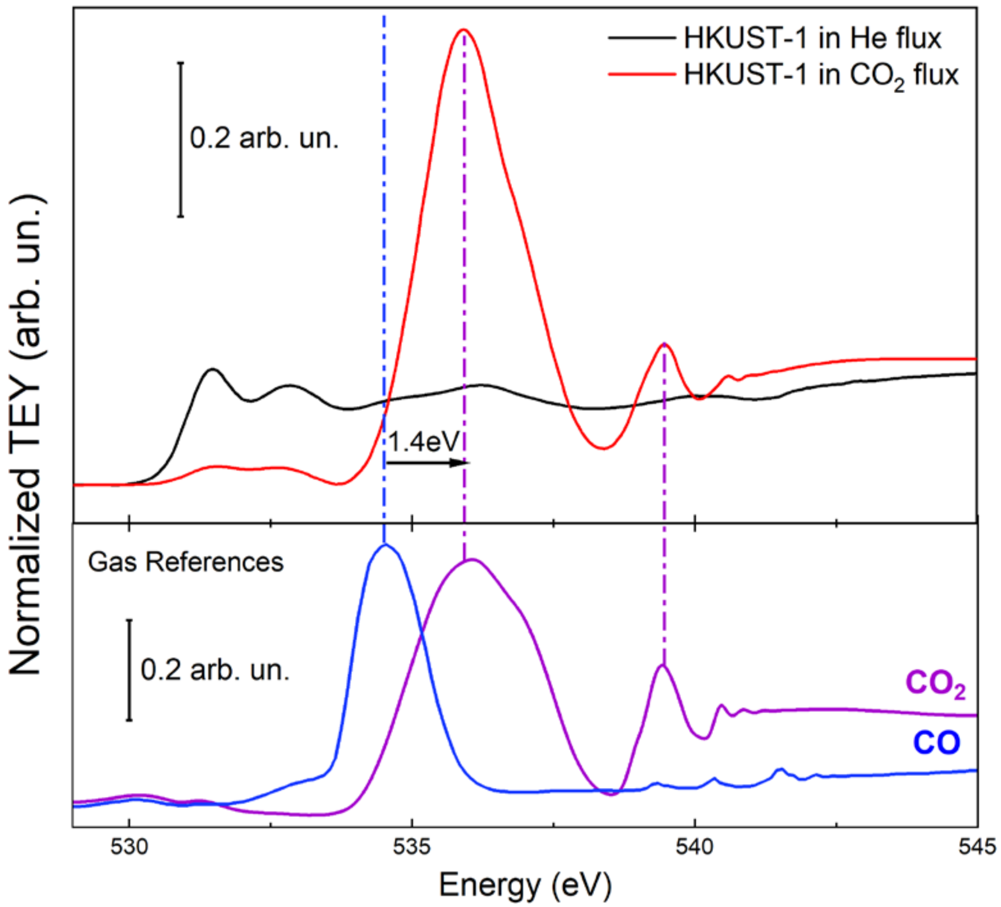

Figure S7. O K-edge spectra of HKUST-1 MOF collected at 160°C in He flux (black curve) and in CO_2 flux (red). In the lower panel the O K-edge spectra of CO (blue) and CO_2 (violet) gases are shown for comparison.

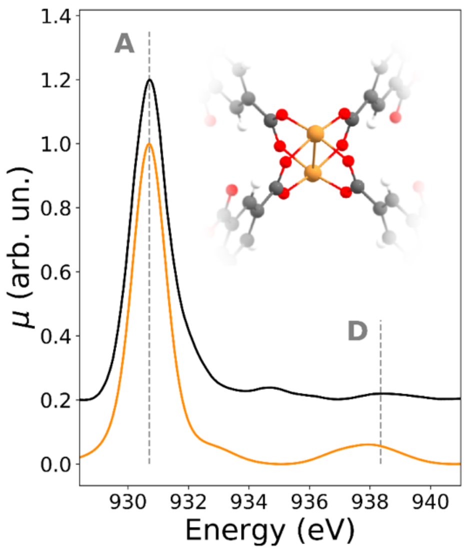

Figure S8. Comparison between the Cu L_3-edge AP-NEXAFS experimental spectrum (black) of HKUST-1 collected at 160°C after exposure to CO_2 and the theoretical spectrum (yellow) calculated for the dehydrated Cu^{2+} dimeric complex. Constant energy cuts (dotted grey lines) are drawn in proximity of the maxima of peaks A and D. The associated molecular cluster is also shown, where the Cu^{2+} cation and the oxygen, carbon and hydrogen atoms are evidenced in orange, red, black and white colors, respectively.

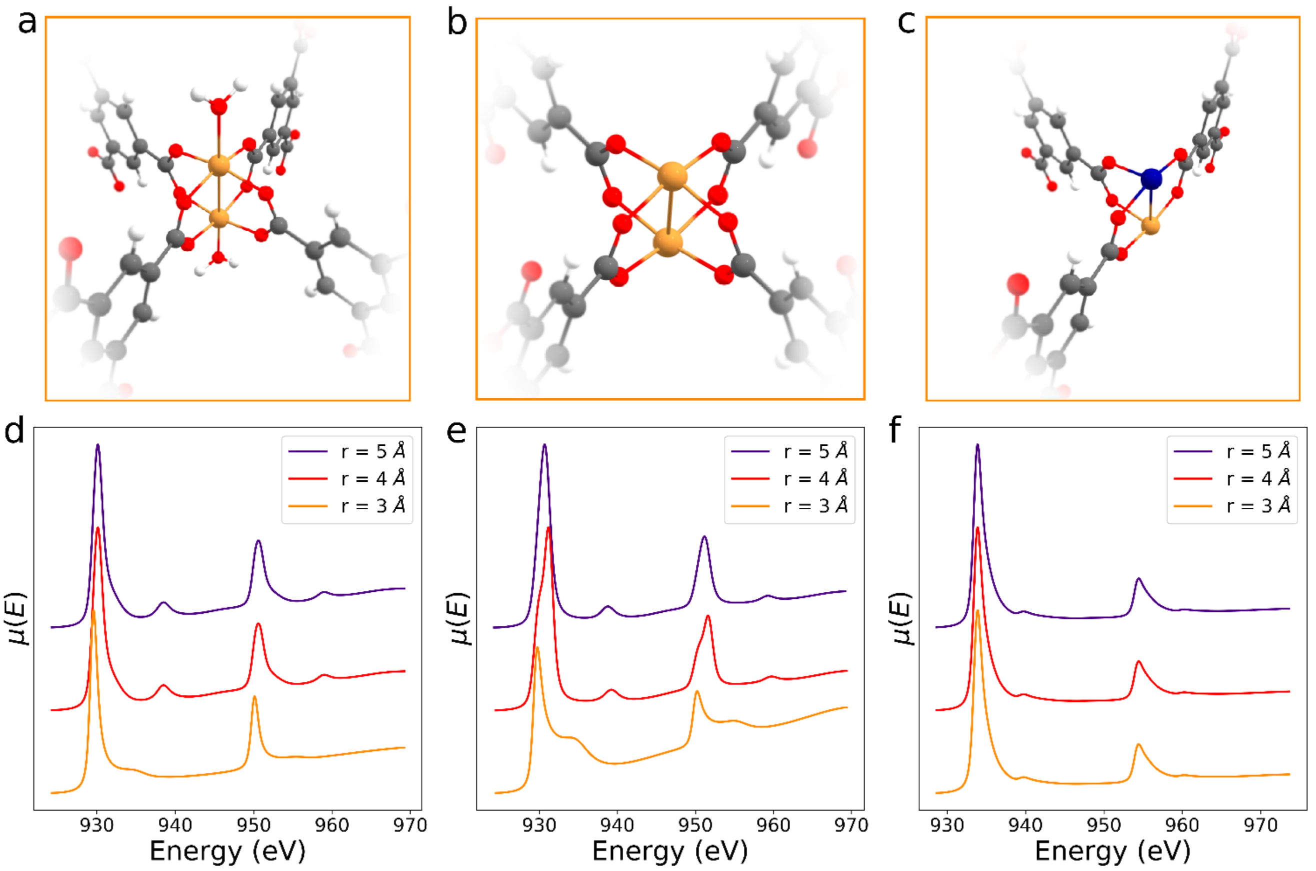

Figure S9. Representative molecular clusters of the hydrated, dehydrated and dehydrated/decarboxylated HKUST-1 MOF dimeric sites (panels a, b and c, respectively) where the Cu^{2+} and Cu^{+} cations are depicted in orange and blue, respectively, while the oxygen, carbon, and hydrogen atoms are depicted in red, gray and white, respectively. The Cu $L_{2,3}$-edge theoretical spectra associated to the dimeric clusters depicted above and calculated within cut-off radii of 3, 4 and 5 Å from the photoabsorber, are shown in panels d, e and f.

Table S1: Structural parameters (atom type, number and coordination distance from the photoabsorber) of the clusters used in the theoretical calculations for Cu_2O, CuO.

	Atom	Coordination Number	Distance (Å)
**Cu_2O**			
	O	2	1.857
	Cu	12	3.032
	O	6	3.555
	Cu	6	4.288
	O	6	4.673
**CuO**			
	O	2	1.950
	O	2	1.961
	O	2	2.784
	Cu	4	2.900
	Cu	4	3.083
	Cu	2	3.173
	O	2	3.408
	Cu	2	3.423
	O	2	3.580
	Cu	2	3.748
	O	2	3.874
	O	2	4.094
	O	2	4.353
	O	2	4.416
	O	2	4.538
	O	2	4.551
	Cu	4	4.667
	Cu	2	4.683
	O	2	4.772
	O	2	4.785
	O	2	4.826
	O	2	4.893

Table S2: Structural parameters (atom type, coordination number and distance from the photoabsorber) of the clusters used in the theoretical calculations of HKUST-1.

	Atom	Coordination Number	Distance (Å)

HKUST-1			
	O	4	1.851
	O	1	2.207
	Cu	1	2.521
	C	4	2.801
	H	2	2.887
	O	4	2.917
	C	4	4.183
	H	4	4.293
	C	4	4.684
	O	1	4.727

EXPERIMENTAL SECTION

Powder X-ray Diffraction (PXRD) Analysis

Gently ground powder of Basolite C300 was deposited in the 2 mm deep hollow of a zero background plate (a properly misoriented quartz monocrystal). The diffraction experiment was performed using Cu-Kα radiation (λ = 1.5418 Å) on a vertical-scan Bruker AXS D8 Advance diffractometer in θ:θ mode, equipped with a Goebel Mirror and a Bruker Lynxeye linear Position Sensitive Detector (PSD), with the following optics: primary and secondary Soller slits, 2.3° and 2.5°, respectively; divergence slit, 0.1°; receiving slit, 2.82°; generator setting: 40 kV, 40 mA. The nominal resolution for the present set-up is 0.08° 2θ (FWHM of the α_1 component) for the LaB_6 peak at about 21.3° (2θ). The HKUST-1 MOF was purchased from Sigma-Aldrich. The accurate diffraction pattern at RT of the sample was acquired in the 5–105° 2θ range, with Δ2θ = 0.02° and exposure time 5 s/step. A Le Bail refinement was carried out in order to check the phase purity within the TOPAS-Academic 6 software.[11] Results of this refinement are reported in Figure S3.

AP-NEXAFS measurements

In order to perform *operando* Ambient Pressure Near edge X-ray absorption fine structure (AP-NEXAFS) measurements, a specially designed reaction cell has been developed at the APE-HE beamline at the ELETTRA synchrotron radiation source.[12,13,14] The samples inside the reactor cell can be heated from RT up to 400°C ca. and they can be exposed to a flux of several gases at the pressure of 1 bar. TEY mode is used to record the experimental spectra by measuring the drain current

from the sample with a picoammeter. The cap of the cell has a Si_3N_4 membrane (100 nm of thickness) that is transparent to X-rays allowing at the same time the vacuum of the beamline to be preserved; the membrane is electrically isolated from the body of the cell where the sample is located. The measurements were performed keeping the sample grounded and applying a positive bias voltage of 40 V to the membrane. The HKUST-1 MOF powder was fixed on a titanium sample holder and pressed in a pit located onto the holder. The cell was mounted in the ultra-high vacuum chamber of the APE-HE beamline, coaxially with the X-ray beam. The experiments were performed collecting the Cu $L_{2,3}$-edge spectra in the energy range 930-948 eV at different temperatures and under different gas fluxes (He, H_2 and CO_2) at 1 bar. Along the gas line a liquid nitrogen trap is placed which eliminates the impurities of water from the gas fluxes. O K-edge spectra were also collected at 160°C in He before and during the CO_2 purge to monitor any oxygen products. The energy calibration was carried out by simultaneously collecting the spectra of the given sample and of a CuO reference. The spectral processing was performed using the THORONDOR software: pre-edge and post-edge spline functions were employed to remove the background and to normalize the spectra.[15] Linear and cubic polynomial functions were used for the pre-edge and for the post-edge extraction, respectively.[16] Ultimately, only for the Cu L_3 edge, the main peak has been normalized to one to enhance the spectral variations during the chemical treatment.

Theoretical XAS calculations

The Cu $L_{2,3}$-edge spectra have been calculated using the Finite Difference Method Near Edge Structure code (FDMNES),[17,18] implementing the recently developed sparse solver method.[19] FDMNES uses the density functional theory (DFT) with a local exchange-correlation potential eventually spin-dependent. The spectra were calculated using the multiple scattering theory (MST) including spin-orbit coupling.[17,18] The muffin-tin (MT) approximation was used for the potential and the MT radii were chosen as to minimize the difference between the potential in the MT spheres and in the interstitial region. The Cu $L_{2,3}$-edge theoretical spectra of CuO and Cu_2O were calculated starting from the corresponding crystallographic structures[20,21] within cut-off radii of 4, 5 and 6 Å from the photoabsorber (Figure S4). The main features of the spectra are well reproduced by using the 5 Å cluster and the more distant atoms have been found to provide an almost negligible contribution.

The theoretical spectra of the hydrated HKUST-1 paddlewheel unit, along with those belonging to the Cu^{2+}/Cu^{2+} and Cu^{+}/Cu^{2+} dimers resulting from dehydration and dehydration/decarboxylation of the pristine unit, were calculated starting from the crystal structure of HKUST-1 hydrated[22] obtained from high-resolution synchrotron powder diffraction, within the MST framework. The dehydrated model was obtaining by removing the apical water molecules, while for the decarboxylated cluster

a DFT optimization was carried out. Different theoretical spectra have been calculated with increasing cut-off radii from the Cu absorber atom (Figure S9) and convergence is achieved at 5 Å.

The Cu^{2+} theoretical spectra were aligned to the experimental data on the basis of the energy of the main L_3-edge peak (peak A, Figure 1a). Conversely, the Cu^+ theoretical spectra were aligned to the Cu^+ peak present in the spectrum of the HKUST-1 sample measured in He (50ml/min, 1bar) at 160°C (peak C, Figure 2a). In order to account for the experimental resolution, a Gaussian broadening of 0.7 eV was applied to all the calculated spectra.

References

(1) Nam, D. H.; Bushuyev, O. S.; Li, J.; De Luna, P.; Seifitokaldani, A.; Dinh, C. T.; García De Arquer, F. P.; Wang, Y.; Liang, Z.; Proppe, A. H.; et al. Metal-Organic Frameworks Mediate Cu Coordination for Selective CO_2 Electroreduction. *J. Am. Chem. Soc.* **2018**, *140* (36), 11378–11386. https://doi.org/10.1021/jacs.8b06407.

(2) Bordiga, S.; Regli, L.; Bonino, F.; Groppo, E.; Lamberti, C.; Xiao, B.; Wheatley, P. S.; Morris, R. E.; Zecchina, A. Adsorption Properties of HKUST-1 toward Hydrogen and Other Small Molecules Monitored by IR. *Phys. Chem. Chem. Phys.* **2007**, *9* (21), 2676–2685. https://doi.org/10.1039/b703643d.

(3) Borfecchia, E.; Maurelli, S.; Gianolio, D.; Groppo, E.; Chiesa, M.; Bonino, F.; Lamberti, C. Insights into Adsorption of NH_3 on HKUST-1 Metal-Organic Framework: A Multitechnique Approach. *J. Phys. Chem. C* **2012**, *116* (37), 19839–19850. https://doi.org/10.1021/jp305756k.

(4) Prestipino, C.; Regli, L.; Vitillo, J. G.; Bonino, F.; Damin, A.; Lamberti, C.; Zecchina, A.; Solari, P. L.; Kongshaug, K. O.; Bordiga, S. Local Structure of Framework Cu(II) in HKUST-1 Metallorganic Framework: Spectroscopic Characterization upon Activation and Interaction with Adsorbates. *Chem. Mater.* **2006**, *18* (5), 1337–1346. https://doi.org/10.1021/cm052191g.

(5) Todaro, M.; Buscarino, G.; Sciortino, L.; Alessi, A.; Messina, F.; Taddei, M.; Ranocchiari, M.; Cannas, M.; Gelardi, F. M. Decomposition Process of Carboxylate MOF HKUST-1 Unveiled at the Atomic Scale Level. *J. Phys. Chem. C* **2016**, *120* (23), 12879–12889. https://doi.org/10.1021/acs.jpcc.6b03237.

(6) Szanyi, J.; Daturi, M.; Clet, G.; Baer, D. R.; Peden, C. H. F. Well-Studied Cu-BTC Still Serves Surprises: Evidence for Facile Cu^{2+}/Cu^{+} Interchange. *Phys. Chem. Chem. Phys.* **2012**, *14* (13), 4383–4390. https://doi.org/10.1039/c2cp23708c.

(7) Wang, W.; Sharapa, D. I.; Chandresh, A.; Nefedov, A.; Heißler, S.; Heinke, L.; Studt, F.; Wang, Y.; Wöll, C. Interplay of Electronic and Steric Effects to Yield Low-Temperature CO Oxidation at Metal Single Sites in Defect-Engineered HKUST-1. *Angew. Chemie Int. Ed.* **2020**, *59* (26), 10514–10518. https://doi.org/10.1002/ANIE.202000385.

(8) Wang, J.; Wang, W.; Fan, Z.; Chen, S.; Nefedov, A.; Heißler, S.; Fischer, R. A.; Wöll, C.; Wang, Y. Defect-Engineered Metal–Organic Frameworks: A Thorough Characterization of Active Sites Using CO as a Probe Molecule. *J. Phys. Chem. C* **2020**, *125* (1), 593–601. https://doi.org/10.1021/ACS.JPCC.0C09738.

(9) Müller, K.; Vankova, N.; Schöttner, L.; Heine, T.; Heinke, L. Dissolving Uptake-Hindering Surface Defects in Metal–Organic Frameworks. *Chem. Sci.* **2018**, *10* (1), 153–160. https://doi.org/10.1039/C8SC03735C.

(10) Heinke, L.; Gu, Z.; Wöll, C. The Surface Barrier Phenomenon at the Loading of Metal-Organic Frameworks. *Nat. Commun. 2014 51* **2014**, *5* (1), 1–6. https://doi.org/10.1038/ncomms5562.

(11) Coelho, A. A. TOPAS and TOPAS-Academic: An Optimization Program Integrating Computer Algebra and

Crystallographic Objects Written in C++. *J. Appl. Crystallogr.* **2018**, *51* (1), 210–218. https://doi.org/10.1107/S1600576718000183.

(12) Castán-Guerrero, C.; Krizmancic, D.; Bonanni, V.; Edla, R.; Deluisa, A.; Salvador, F.; Rossi, G.; Panaccione, G.; Torelli, P. A Reaction Cell for Ambient Pressure Soft X-Ray Absorption Spectroscopy. *Rev. Sci. Instruments* **2018**, *89*, 54101. https://doi.org/10.1063/1.5019333.

(13) Fracchia, M.; Ghigna, P.; Pozzi, T.; Anselmi Tamburini, U.; Colombo, V.; Braglia, L.; Torelli, P. Stabilization by Configurational Entropy of the Cu(II) Active Site during CO Oxidation on Mg0.2Co0.2Ni0.2Cu0.2Zn0.2O. *J. Phys. Chem. Lett.* **2020**, *11* (9), 3589–3593. https://doi.org/10.1021/acs.jpclett.0c00602.

(14) Braglia, L.; Fracchia, M.; Ghigna, P.; Minguzzi, A.; Meroni, D.; Edla, R.; Vandichel, M.; Ahlberg, E.; Cerrato, G.; Torelli, P. Understanding Solid-Gas Reaction Mechanisms by Operando Soft X-Ray Absorption Spectroscopy at Ambient Pressure. *J. Phys. Chem. C* **2020**, *124* (26), 14202–14212. https://doi.org/10.1021/acs.jpcc.0c02546.

(15) Calvin, S. *XAFS for Everyone*; CRC Press, 2013. https://doi.org/10.1201/b14843.

(16) Simonne, D. H.; Martini, A.; Signorile, M.; Piovano, A.; Braglia, L.; Torelli, P.; Borfecchia, E.; Ricchiardi, G. *THORONDOR* : A Software for Fast Treatment and Analysis of Low-Energy XAS Data. *J. Synchrotron Radiat.* **2020**, *27* (6). https://doi.org/10.1107/S1600577520011388.

(17) Joly, Y. X-Ray Absorption near-Edge Structure Calculations beyond the Muffin-Tin Approximation. *Phys. Rev. B - Condens. Matter Mater. Phys.* **2001**, *63* (12), 125120. https://doi.org/10.1103/PhysRevB.63.125120.

(18) Bunău, O.; Joly, Y. Self-Consistent Aspects of x-Ray Absorption Calculations. *J. Phys. Condens. Matter* **2009**, *21* (34), 11. https://doi.org/10.1088/0953-8984/21/34/345501.

(19) Guda, S. A.; Guda, A. A.; Soldatov, M. A.; Lomachenko, K. A.; Bugaev, A. L.; Lamberti, C.; Gawelda, W.; Bressler, C.; Smolentsev, G.; Soldatov, A. V.; et al. Optimized Finite Difference Method for the Full-Potential XANES Simulations: Application to Molecular Adsorption Geometries in MOFs and Metal-Ligand Intersystem Crossing Transients. *J. Chem. Theory Comput.* **2015**, *11* (9), 4512–4521. https://doi.org/10.1021/acs.jctc.5b00327.

(20) Åsbrink, S.; Norrby, L.-J. A Refinement of the Crystal Structure of Copper(II) Oxide with a Discussion of Some Exceptional e.s.d.'s. *Acta Crystallogr. Sect. B* **1970**, *26* (1), 8–15. https://doi.org/10.1107/S0567740870001838.

(21) Kirfel, A.; Eichhorn, K. Accurate Structure Analysis with Synchrotron Radiation. The Electron Density in Al2O3 and Cu2O. *Acta Crystallogr. Sect. A* **1990**, *46* (4), 271–284. https://doi.org/10.1107/S0108767389012596.

(22) Yakovenko, A. A.; Reibenspies, J. H.; Bhuvanesh, N.; Zhou, H.-C. Generation and Applications of Structure Envelopes for Porous Metal–Organic Frameworks. *urn:issn:0021-8898* **2013**, *46* (2), 346–353. https://doi.org/10.1107/S0021889812050935.
